# Supplementary material for: Barriers and facilitators to HIV testing among transgender people in Georgia: Qualitative study results using the COM-B Framework
Source: PLOS Glob Public Health. 2026 Mar 6;6(3):e0005819. doi: 10.1371/journal.pgph.0005819 (PMC12965578; doi:10.1371/journal.pgph.0005819)
Supplement: S1 Interview Guide — (DOCX) [file pgph.0005819.s001.docx]

**Semi-structured interview guide used for in-depth Interviews among transgender people (trans woman, trans-man, non-binary)**

[Interviewer’s note: *Following an explanation of the study objectives and participation procedures, and after securing written informed consent from the participant, the interviewer will start the audio recording to prepare transcripts needed for the detailed analyses*.]

**Introduction**

Thank you for agreeing to take part in this in-depth interview. You’ve been informed about the study’s goals, as well as the guidelines and rights associated with your participation. We have also obtained your written consent. We’re now ready to get started.

You are informed that the study's research interests are to identify the barriers and facilitators to HIV testing among transgender individuals, including examining personal experiences with testing services, the role of stigma, social and policy influences, as well as the effectiveness of digital tools, awareness campaigns, and potential strategies to improve access to testing services.

Prior to this study, we have carried out quantitative study results, that revealed that a high HIV burden among transgender individuals (TG) in Georgia, particularly transgender women. This is combined with significant gaps in the utilization of HIV prevention services - HIV testing in the past six months were reported by 76.8% of participants and predictors for testing were living alone and experiences of enacted stigma. Based on this findings further research is needed to explore the intersection of stigma and other barriers affecting a low uptake of HIV testing to inform the development of effective targeted interventions.

Participation. All of your views are important. There aren’t any right or wrong answers. Please be as open as possible in telling me what you think, whether it’s positive or negative.

I want to remind you that our interview is expected to last around 60-90 minutes. Some questions may feel sensitive or personal, and you are completely free to skip any questions that make you uncomfortable or to withdraw from the interview at any time. This interview will be audio-recorded for transcription and analysis purposes, and your confidentiality will be fully protected.

Confidentiality/anonymity and honesty. Before starting I just want to assure you that even though we’re recording this, no names will be connected with anything said in this interview (in this group), and no names will be used in any analyses, reports or papers. Please feel comfortable in expressing your opinions - nothing you say will be connected with you personally. Also, nothing that is said in this session should be repeated to anyone else with connection to any names.

If you have any questions or concerns about the process, please share them with me now. Additionally, I kindly ask you to reaffirm your consent to participate before we begin.

|  | |
| --- | --- |
| **About Framework** | The questions are prepared according to COM-B (Capability, Opportunity, Motivation and Behavior) framework to understand a specific behavior occurs and how to create targeted interventions that lead to effective change.  **Capability** refers to an individual’s psychological and physical ability to take HIV test.  **Opportunity** refers to external factors that make a HIV testing possible.  **Motivation** refers to the conscious and unconscious cognitive processes that **influence and drive** a person's decision to get tested for HIV. It shapes their willingness and actions toward seeking an HIV test. |
| **Sociodemographic Characteristics and introductory questions:**  [*Interviewer note: Before the interview starts, the interviewer will make notes about the following sociodemographic information*]   - - *Age*   - *City and place of residence (living in a shelter?)*   - *Have you ever applied for social services (except for medical care)? Please specify*   ***Living Situation***  Can you tell me about your current living situation? For example, do you live in a permanent residence, shared housing, or a shelter?  ***Employment and Financial Stability***   - - How would you describe your current employment status and financial situation? (Prompt if needed: Are you currently working? Do you have a stable source of income?)   ***Dependents***   - - Are there any people who financially depend on you, or whom you support in your daily life?   ***TG Needs***   - - From your perspective, what are the most urgent social needs for transgender people in Georgia today? (Prompt if needed: housing, employment, food, clothing, legal documents, health services, or other types of support?) | |
| **Use of prevention/ HIV testing services** **Personal Use of HIV Prevention Services** Capability (Physical & Psychological)  - Can you tell me about your experiences using HIV prevention services? Please tell me more about that. - What kinds of HIV prevention services have you used, such as condoms, lubricants, PrEP, or PEP? How was that experience for you? - How do you feel about the way transgender people in Georgia are able to access HIV prevention services? - → Can you tell me more about what makes it easy or difficult for them?   Opportunity (Social & Physical)   - Among the transgender people you know, how common is it to use prevention services? → *What makes some people more likely to use them?*  **HIV Testing Experiences** (Capability & Motivation)**Experience and frequency of HIV testing**   - Can you walk me through the last time you were tested for HIV? What led up to that moment? - How often do you think transgender people in your circle tend to get tested for HIV? → What might influence how often they test?  **Self-Testing Accessibility**   - Have you ever used an HIV self-test? If yes, how did you receive it? (e.g., brought by a friend, partner, or outreach worker) **(Opportunity)** - How acceptable is it for you/ your sexual partner? *What felt good about it? What felt difficult?* **(Motivation)**  **Barriers to HIV Testing****General Barriers** (Opportunity & Motivation)  - In your opinion, what are some of the main things that make it hard for transgender people to get tested for HIV in Georgia?  → Can you think of an example or a time when this happened?  **Lack of Information or Awareness (Capability)**  - How well do you think transgender people are informed about the risks of HIV infection? - How do you think if there is a lack of knowledge about where HIV testing/HIV self-testing services in the country? - → Where do people usually learn about it?  **Fear and Stigma (Opportunity)**  - Have you ever felt hesitant to get tested for HIV due to fear of receiving a positive result? Would you explain that further? - What are some concerns you’ve heard from others about HIV testing and confidentiality? - In your view, how does stigma related to gender identity or HIV affect people’s decisions to get tested for HIV? Would you say it facilitates or hinders testing — and in what ways? - →Have you heard or experienced anything like that? - Do you think living alone influences HIV testing behaviors among transgender people? Would you say it facilitates or hinders testing — and why? - →Have you heard or experienced anything like that?  **Access and Convenience** (Opportunity)  - Do you think the availability of HIV testing sites / HIV self-testing is a challenge in Georgia? (Prompt if needed: Are there enough testing sites, or are they located too far away?) - →if yes, please tell me what types of practical issues—like transport, cost, or waiting times—have you or others experienced?  **Personal Experience and stories** (Motivation and Opportunity)  - Can you share an experience where you or someone you know was treated unfairly or judged at a health service because of gender identity in Georgia? - Have you had a moment where something made you decide not to get tested? *Please specify such episodes incurred where? by who? How often? Etc.? Any other thoughts/explanations?* - If it’s been over 6 months since your last test, what have been some of the reasons?  **Facilitators to HIV Testing****Factors Influencing HIV testing/Service Use**  **Who Gets Tested** (Motivation)   - In your opinion, which transgender people are most likely to use HIV testing/ prevention services? (Prompt if needed: TG with low-income; transgender women; more vulnerable; those involved in sex work; enacted stigma due to gender identity; live alone) - What do you think motivates these individuals to seek prevention and testing services?   ***Personal Motivators***   - What helped or motivated you to get tested for HIV? (if any) (Motivation) - How has your understanding of HIV risk influenced your decisions to get tested? (Motivation) - Are you aware that HIV testing/HIV self-testing services in Georgia are free, anonymous, and confidential? **(Capability)** - →*If yes, where do you get information about HIV testing services?* **(Opportunity)** - Have you ever come across a campaign or post that made you stop and think about getting tested? →*If yes, c*an you elaborate more on that **(Capability & Opportunity)**  **Raising Awareness and Access to Information**   - How do you think, where do your transgender peers usually get information about HIV testing/HIV self-testing services? **(Opportunity)** - Do you think understanding personal risks encourages other transgender people to seek HIV testing? *c*an you elaborate more on that **(Motivation)** - Did you see any social media posts/ HIV testing campaign about HIV testing or self-testing that encouraged you or someone you know to get tested? Please describe this experience **(Opportunity and Motivation)**  **Influence of Others****Influence of Outreach Workers and Campaigns (Opportunity and Motivation)**  - Have you ever been tested for HIV because an outreach worker offered testing (in the field or at an office)? What was that like for you?  **Social Influences (Opportunity and Motivation)**  - Has anyone—a friend, partner, peer, or outreach worker—encouraged you to get tested? What was that like for you? - If your partner tested positive for HIV, was this a key factor in your decision to get tested?  **Economic Influences (Opportunity and Motivation)**  - Can you talk about how money or other resources affect whether transgender people get tested for HIV? → *Do you have any personal experiences you’d be comfortable sharing*? - What kinds of support (financial or otherwise) would make it easier for people to access testing? → For example, what do you think about things like taxi money, food or telephone vouchers, or other support? | |
| **Final Reflections**  - Is there anything else you’d like to share about this topic that we haven’t talked about? | |

Now, we are concluding our interview. Thank you for your valuable participation. If you have any questions or concerns, please contact me any time via email or phone.
