# Supplementary material for: Barriers and facilitators to HIV testing among transgender people in Georgia: Qualitative study results using the COM-B Framework
Source: PLOS Glob Public Health. 2026 Mar 6;6(3):e0005819. doi: 10.1371/journal.pgph.0005819 (PMC12965578; doi:10.1371/journal.pgph.0005819)
Supplement: S1 Codebook — (DOCX) [file pgph.0005819.s002.docx]

S2. Codebook: COM-B Constructs, Sub-Constructs, and Main Themes

| **COM-B construct** | **COM-B sub-construct** | **Main theme** |
| --- | --- | --- |
| Capability | Physiological Capability | Awareness/knowledge of HIV transmission risks |
|  |  | Awareness of free and anonymous services |
|  |  | Lack of awareness about free and anonymous services |
|  |  | Lack of education and limited sexual health literacy |
| Opportunity | Physical Opportunity | Good access to HIV testing sites, including outreach based HIV testing and oral self- testing |
|  |  | Social media and dating apps |
|  |  | Geographic barriers |
|  | Social Opportunity | Community encouragement and support |
|  |  | Social stigma, discrimination, violence |
|  |  | Social stigma, discrimination, violence |
|  |  | Fear of breach of confidentiality |
|  |  | Poor communication |
| Motivation | Reflective Motivation | Recognition of HIV testing as part of self-care and personal health |
|  |  | Moral duty to community |
|  |  | Living alone |
|  |  | High HIV risk perception |
|  |  | Living alone |
|  |  | Low perceived need and lack of interest in HIV testing |
|  |  | Fear of getting positive HIV test results |
|  | Automatic Motivation | Incentives (financial and non-financial) |
|  |  | Substance use, trauma, mental health |
