# Supplementary material for: Barriers and facilitators to HIV testing among transgender people in Georgia: Qualitative study results using the COM-B Framework
Source: PLOS Glob Public Health. 2026 Mar 6;6(3):e0005819. doi: 10.1371/journal.pgph.0005819 (PMC12965578; doi:10.1371/journal.pgph.0005819)
Supplement: S1 COREQ Checklist — (DOCX) [file pgph.0005819.s003.docx]

**S3 COREQ Check list.** COREQ: Consolidated Criteria for Reporting Qualitative Research

**Domain 1: Research team and reflexivity**

1. **Interviewer/facilitator**
   Interviews were conducted by trained qualitative researcher Marine Gogia (main researcher of the study).
2. **Credentials**
   Interviews were conducted by researcher with advanced training in medicine and public health (MD, MPH), including doctoral-level training (PhD candidate), and experience in qualitative research.
3. **Occupation**
   Researchers were employed in public health research and community-based HIV prevention.
4. **Gender**
   Female.
5. **Experience and training**
   Interviewers had prior experience conducting qualitative interviews with key populations, including transgender communities.
6. **Relationship established**
   No prior personal relationship existed between interviewers and participants.
7. **Participant knowledge of the interviewer**
   Participants were informed about the study objectives, the researchers’ institutional affiliations, and the voluntary nature of participation.
8. **Interviewer characteristics**
   Researcher was aware of potential power dynamics and aimed to create a safe, respectful, and non-judgmental interview environment.

**Domain 2: Study design**

1. **Methodological orientation**
   Qualitative exploratory study using thematic analysis informed by the COM-B model.
2. **Sampling**
   Purposive sampling with support from community-based organizations.
3. **Method of approach**
   Participants were approached by social workers at partner organizations and invited to participate.
4. **Sample size**
   15 participants.
5. **Non-participation**
   Two individuals initially agreed but later declined; replacements were recruited.
6. **Setting of data collection**
   Interviews were conducted remotely via Zoom.
7. **Presence of non-participants**
   No non-participants were present during interviews.
8. **Description of sample**
   Participants self-identified as transgender women, transgender men, or non-binary individuals, aged 18–45, HIV-negative.
9. **Interview guide**
   A semi-structured interview guide informed by the COM-B framework was used (included as Supporting Information).
10. **Repeat interviews**
    No repeat interviews were conducted.
11. **Audio/visual recording**
    All interviews were audio-recorded with participants’ consent.
12. **Field notes**
    Researcher documented reflections and early impressions during familiarization.
13. **Duration**
    Interviews lasted approximately one hour.
14. **Data saturation**
    Sampling continued until thematic saturation was reached, defined as no new themes emerging in later interviews.

**Domain 3: Analysis and findings**

1. **Number of data coders**
   Two researchers independently coded the data: Marine Gogia and Tamar Zurashvili (MD, MPH, PhDc).
2. **Description of the coding tree**
   Codes were developed inductively and organized deductively under COM-B domains; the final codebook is provided as Supporting Information (S2).
3. **Derivation of themes**
   Themes were derived through iterative comparison, discussion, and refinement.
4. **Software**
   Manual coding was employed.
5. **Participant checking**
   Member checking was not conducted.
6. **Quotations presented**
   Representative participant quotes are included to illustrate each theme.
7. **Data and findings consistent**
   Findings are grounded in participants’ narratives and supported by verbatim quotes.
8. **Clarity of major themes**
   Major themes and sub-themes are clearly described in the Results section.
9. **Clarity of minor themes**
   Less frequent perspectives are explicitly reported using qualitative frequency descriptors.
10. **Overall transparency**
    The analytic process and theoretical mapping are described in detail to ensure rigor and transparency.
