# Supplementary material for: Barriers and facilitators to HIV testing among transgender people in Georgia: Qualitative study results using the COM-B Framework
Source: PLOS Glob Public Health. 2026 Mar 6;6(3):e0005819. doi: 10.1371/journal.pgph.0005819 (PMC12965578; doi:10.1371/journal.pgph.0005819)
Supplement: Attachment — Submitted filename: Response to Reviewers.docx [file pgph.0005819.s005.docx]

### Submitted Paper Title: Barriers and Facilitators to HIV Testing among Transgender People in Georgia: Qualitative Study Results Using the COM-B Framework (PGPH-D-25-02845)

**Response to Reviewers**

**Journal Requirements:**

| **Review Comments to the Author** | **Responses to the comments/questions** |
| --- | --- |
| 1. Please amend your detailed Financial Disclosure statement. This is published with the article. It must therefore be completed in full sentences and contain the exact wording you wish to be published.   i. State what role the funders took in the study. If the funders had no role in your study, please state: “The funders had no role in study design, data collection and analysis, decision to publish, or preparation of the manuscript.” | Thank you for the comment. We have updated the Financial Disclosure statement to clarify that the funders had no role in the study design, data collection and analysis, decision to publish, or preparation of the manuscript. The Funding statement now reads as follows:  *“The study was supported by the Fogarty International Center and the National Institute on Alcohol Abuse and Alcoholism of the National Institutes of Health (Grant D43TW011532 to MD).* ***Funding acquisition was led by MD. The funders had no role in the study design, data collection and analysis, decision to publish, or preparation of the manuscript****”.* |
| 2. Please ensure that your Ethics Statement is available in its entirety at the beginning of your Methods section, under a subheading 'Ethics Statement'. | We have revised the manuscript to include the complete Ethics Statement at the beginning of the Methods section under the subheading “Ethics Statement.” |
| 3. We note that you have included your Figures within the body of your manuscript. Please remove the Figures from the body of your manuscript and upload them as separate Figure files. | We have removed the Figures from the main manuscript text and submitted each Figure as a separate file, as requested. In addition, we will submit all figure files in compliance with PLOS requirements and will use the PLOS NAAS tool to verify formatting, resolution, and file type prior to final submission. |
| 4. Tables should not be uploaded as individual files. Please remove these files and include the Tables in your manuscript file as editable, cell-based objects. For more information about how to format tables, see our guidelines:   <https://journals.plos.org/globalpublichealth/s/tables> | The table was previously included in the manuscript and also uploaded as a separate file. We have now removed the separate file and retained the table only within the main manuscript as an editable, cell-based table, in accordance with the journal’s formatting guidelines. |
| 5. We have noticed that you have uploaded Supporting Information files, but you have not included a list of legends. Please add a full list of legends for your Supporting Information files after the references list. | We have added a complete list of legends for all Supporting Information files after the References section, as requested. |
| 6. In the online submission form, you indicated that “Due to the sensitive nature of the interviews conducted with transgender and other key populations, and because all interviews and transcripts are in Georgian, full transcripts cannot be made publicly available in order to protect participant confidentiality. De-identified, aggregated data supporting the findings of this study, are available from the corresponding author upon reasonable request and subject to approval by the National Center for Disease Control and Public Health Ethics Committee.”   All PLOS journals now require all data underlying the findings described in their manuscript to be freely available to other researchers, either  1. In a public repository,  2. Within the manuscript itself, or  3. Uploaded as supplementary information.  This policy applies to all data except where public deposition would breach compliance with the protocol approved by your research ethics board. If your data cannot be made publicly available for ethical or legal reasons (e.g., public availability would compromise patient privacy), please explain your reasons by return email and your exemption request will be escalated to the editor for approval. Your exemption request will be handled independently and will not hold up the peer review process, but will need to be resolved should your manuscript be accepted for publication. One of the Editorial team will then be in touch if there are any issues. | Due to the sensitive nature of interviews conducted with transgender people and other key populations, and to protect participant confidentiality, the full qualitative interview transcripts cannot be made publicly available. In addition, all interviews and transcripts are in Georgian, increasing the risk of deductive disclosure if shared publicly.  De-identified, aggregated data supporting the findings of this study are available upon reasonable request and subject to ethical approval by the Institutional Review Board of the National Center for Disease Control and Public Health (NCDC), Georgia. Data access requests should be directed to the NCDC Institutional Review Board (contact: Marina Topuridze; email: irb.ncdc@gmail.com; m.topuridze@ncdc.ge).  We respectfully request an exemption from public deposition of full interview transcripts on ethical grounds. |
| 7. **Methodology** The methodology section lacks a clear articulation of the study design. While the theoretical framework is mentioned, the overall study design is not explicitly stated. I appreciate that the COM-B model informed the development of the semi-structured interview guide, as indicated in the interview guide. However, the description of the framework underpinning the interview guide should be incorporated into the main manuscript rather than remaining solely in the guide. Including this information in the manuscript would help clarify the conceptual meaning of each COM-B domain and strengthen the theoretical transparency and interpretability of the study. | Thank you for this comment. In response, we have revised the Methodology section to clearly describe the overall study design as a qualitative study using in-depth interviews. We have also incorporated a detailed description of the COM-B framework into the main manuscript, including definitions of its three domains (Capability, Opportunity, and Motivation) and an explanation of how the framework informed the development of the semi-structured interview guide, data collection, and thematic analysis. These revisions strengthen the study’s conceptual clarity, theoretical transparency, and interpretability. The Study design section now reads as follows:  *“This study employed a qualitative exploratory design using in-depth interviews to examine barriers and facilitators to HIV testing among transgender individuals in Georgia. The study was guided by the COM-B model, a widely used behavior change framework that conceptualizes behavior as the interaction between individuals’ Capability, Opportunity, and Motivation to perform a given behavior [12]. COM-B was selected because HIV testing represents a complex health behavior influenced by individual knowledge and beliefs, social and structural contexts, and motivational processes—factors that are particularly salient for transgender populations facing stigma and access barriers. In this study, the COM-B framework provided a structured lens to explore how personal capacities, environmental conditions, and motivational drivers shape HIV testing decisions among transgender individuals in Georgia, and it informed the development of the semi-structured interview guide, the thematic analysis, and the interpretation of findings”*. |
| 8. **Study setting** Naming community-based organisations where TG individuals were recruited is not inherently unethical, but it is often unnecessary and potentially risky. Unless strong justification and explicit consent are provided, anonymising organisations is generally the more ethical approach. | We have revised the Study Setting section to anonymize the participating community-based organizations. This change was made to reduce potential risks to organizations and communities operating in a sensitive and increasingly restrictive sociopolitical environment, while preserving essential contextual information relevant to the study. |
| 9. **Participants and Recruitment** The authors do not clearly explain how the sample size of 15 participants was determined. Please clarify the rationale for this sample size, including whether it was guided by methodological considerations such as data saturation, feasibility constraints, prior qualitative research, or established qualitative sampling principles. The manuscript would benefit from clarification regarding the inclusion criteria related to participants’ HIV status. Specifically, please explain the rationale for including only HIV-negative individuals and excluding those with unknown HIV status, given that the study’s objective also focused on barriers to HIV testing behaviors. Additionally, this restriction should be acknowledged as a limitation, as it may have influenced the findings. | We have revised the “Participants and Recruitment” section to clarify the rationale for the sample size, inclusion criteria, and their potential implications. We also added that the study aimed to include all gender-diverse sub-groups and acknowledged that restricting the sample to HIV-negative participants may limit the generalizability of the findings to transgender individuals with unknown or positive HIV status.  In line with your suggestion, we have also revised the Study Limitations section to include the following statement: *“In addition, the inclusion of only HIV-negative participants may limit the generalizability of findings to all transgender individuals, particularly those with unknown or positive HIV status, and could have influenced the barriers and facilitators identified.”* |
| 10. **Data collection** The manuscript would benefit from clarification regarding the pilot phase. Please specify whether the pilot was conducted with the same participants included in the main study or with a separate group. The manuscript does not specify the form of informed consent obtained from participants. Please clarify whether consent was written or verbal, and briefly describe how consent was documented. | Thank you for this comment. We have clarified the Data Collection section to specify that the pilot phase was conducted with two participants from a separate group who were not included in the main study, allowing refinement of the interview guide without influencing responses of study participants. We also added details on informed consent, specifying that verbal consent was obtained prior to each interview after providing participants with a clear explanation of the study objectives, topics, potential risks and benefits, and their right to withdraw at any time. The revised Data Collection section now reads as follows:  *“The semi-structured interview guide was pilot-tested with two participants from a separate group prior to the main study to assess clarity, comprehensiveness, and relevance. Feedback from these pilot interviews was used to refine the guide and ensure alignment with the study objectives. Data collection was conducted exclusively via Zoom between February and April 2025. Interviews lasted an average of one hour, and all participants received 70 Georgian Lari (GEL; approximately USD 25) as an incentive. Verbal informed consent was obtained from each participant prior to the interview. The consent process included a detailed explanation of the study objectives, topics to be discussed, potential risks and benefits, and participants’ right to withdraw at any time.”* |
| 11. **Analysis** The authors do not clearly define the COM-B sub-constructs used in the analysis. The absence of these definitions makes it difficult to assess how the framework was applied and to evaluate the methodological rigour of the study. Please explicitly define each COM-B sub-construct and explain how they informed data collection and analysis to improve clarity and transparency. | Thank you to the reviewer for this comment. In response, we have revised the Methods (Analysis) section to explicitly define each COM-B sub-construct—Capability (psychological and physical), Opportunity (social and physical), and Motivation (reflective and automatic). We have also clarified how these sub-constructs informed the development of the semi-structured interview guide, coding procedures, thematic analysis, and interpretation of findings. These revisions enhance transparency and allow readers to clearly understand how the COM-B framework was applied in this study. The revised Analysis section now reads as follows:  ‘*’Data were analyzed using thematic analysis informed by the COM-B model to explore barriers and facilitators to HIV testing among transgender (TG) participants. Interviews were audio-recorded, transcribed verbatim, and read repeatedly to ensure familiarity with the data. During the initial phase, two researchers independently reviewed the transcripts, noted recurring patterns and preliminary impressions, and conducted manual open coding by labeling relevant text segments reflecting participants’ experiences, beliefs, and behavioral influences. Through iterative discussions*, *codes were compared, refined, and organized into preliminary themes, resulting in a shared codebook.*  *In the subsequent phase, emerging themes were organized deductively under the COM-B model’s core domains: Capability, Opportunity, and Motivation. Capability encompassed both psychological and physical capability (e.g., HIV knowledge, health literacy, and awareness of services). Opportunity included physical opportunity (e.g., access, geographic factors, outreach-based testing, and HIV self-testing) and social opportunity (e.g., stigma, confidentiality, and community support). Motivation was examined across reflective motivation (e.g., risk perception, self-care, and moral responsibility) and automatic motivation (e.g., fear, incentives, substance use, and trauma). This explicit mapping of codes and themes to COM-B sub-constructs ensured transparent application of the theoretical framework and strengthened methodological rigor (S2 Codebook).”* |
| 12. The manuscript does not include a section addressing trustworthiness. Please add a dedicated section describing how trustworthiness was enhanced in the study. | Thank you for this comment. In response, we have added a dedicated Trustworthiness section to the Methods, detailing the strategies used to enhance methodological rigor. These include independent coding by two researchers, iterative discussions to refine and agree on the codebook, explicit mapping of themes to the COM-B framework, and the use of verbatim participant quotations to anchor interpretations in the data. The Trustworthiness section now reads as follows:  *“Trustworthiness was enhanced through multiple strategies, including independent coding by two researchers, iterative discussions to refine the codebook, explicit mapping of themes to the COM-B framework, and the use of verbatim participant quotes to ground interpretations in the data.”* |
| 13. Results The manuscript would benefit from clarification on how participants were identified as trans men, trans women, or non-binary. Please specify whether these identities were self-reported by participants and describe the process or criteria used to categorise gender identity. | Thank you for your comment. We have clarified in the Results section that participants’ gender identities were self-identified. The Participant Characteristics subsection now reads as follows:  “A total of 15 transgender and non-binary individuals participated in this study. Among them, eight **self-identified** as transgender women, five as non-binary, and two as transgender men. Participants ranged in age from 18 to 45 years, with a median age of 23 years and a mean age of 26 years.” |

**Reviewer #1**

| 14. The COM-B model needs explanation, both overall as a theoretical framework, and how it informed the design of the semi-structured interview guide and analysis. In addition, it would be helpful to know how each of the domains of COM-B are conceptualized in this context and why this was chosen as the theory of behavior change for this study. I think this could either be provided in the introduction or the methods. | Thank you for this comment. We have revised the **Study Design** and **Analysis** sections (modified text is provided above) to provide a clearer explanation of the COM-B model as the theoretical framework underpinning the study and to better articulate its relevance. As described above, we now explicitly define the COM-B domains and sub-constructs and clarify how the framework informed the development of the interview guide, data analysis, and interpretation of findings. |
| --- | --- |
| 15. I encourage the authors to provide their final interview guide as a supplemental file. | Thank you for this suggestion. The final semi-structured interview guide has now been added as Supporting Information (S1). |
| 16. Further justification for the sample size is needed. Was theoretical or meaning saturation achieved? | Thank you for your comment. We have clarified in the Participants and Recruitment section that the target sample size of 15 participants was determined based on anticipated data saturation and feasibility constraints within the study timeframe. Recruitment continued until no new themes emerged, indicating that thematic saturation was achieved. We also clarified that the study aimed to include all gender-diverse sub-groups, and that two participants who initially agreed later declined and were replaced. Revised **Participants and Recruitment** looks now as follows:  *“We aimed to include all gender-diverse sub-groups in the study (transgender women, non-binary individuals, and transgender men). The target sample size of 15 participants was determined based on anticipated data saturation and feasibility constraints within the study timeframe. Recruitment continued until no new themes emerged, indicating that thematic saturation was achieved. Two individuals initially agreed to participate but later declined; replacements were recruited.”* |
| 17. The analysis section is generally clear, but additional information is needed, including the make-up of the coding team, whether and how qualitative analysis software was used, and how the codebook was developed and finalized (this is hinted at, but needs to be more explicit). It would also be helpful to include the final codebook as a supplemental file. | The Analysis section has been revised (**questions 11 and 12 above**) to clarify that two researchers independently conducted open coding, followed by iterative discussions to develop the codebook. Manual coding was employed, and codes were mapped deductively to the COM-B sub-constructs. The process for refining and finalizing the codebook is now explicitly described. The final codebook has been added as Supporting Information (S2). |
| 18. I appreciate the authors providing an introduction to the results and themes, but the figures made it difficult to understand the key themes vs. sub-themes, and as a result this introduction to the result was not as helpful for the reader’s understanding as it could be. | Thank you for the comment. The Results section has been revised to improve clarity by removing the figures from the main text and enhancing the textual description of themes and sub-themes, making the introduction to the results clearer and more informative for the reader. |
| 19. The tables are also somewhat bulky and difficult to understand, and there are so many quotes that the voices of the participants get a little lost, I think. I think re-organizing the results by theme instead of by COM-B construct would be clearer. | Thank you for your suggestions. We appreciate the point about organizing results by theme for clarity. As our study was guided by the COM-B framework, we structured the results according to its constructs to maintain alignment with the theoretical model. At the same time, we recognize the importance of thematic clarity, which is addressed in the Figures, where barriers and facilitators to HIV testing are visually summarized according to COM-B constructs in a clear and accessible format.  We revised the opening of the Results section to explicitly clarify how findings are presented:  *“Barriers and facilitators to HIV testing among TG participants are presented within the COM-B framework (Capability, Opportunity, and Motivation). An overview of all identified barriers and facilitators is provided in Table 1, while their distribution across COM-B domains is shown in Figure 1 (Barriers to HIV testing according to COM-B) and Figure 2 (Facilitators to HIV testing according to COM-B). The narrative Results below describe these findings within each COM-B domain.”*  Regarding the table 1, we agree that the original version was dense and could be difficult to navigate. To improve clarity and readability, we have revised the tables as follows:   1. Quotes are now grouped under broader themes rather than listed individually, reducing table length and allowing readers to quickly identify the main barriers and facilitators. 2. The number of supporting quotes has been reduced from 70 to 41, selecting the most illustrative examples for each theme while retaining participants’ voices. 3. Links to the COM-B constructs have been maintained, so readers can clearly see how themes relate to capability, opportunity, and motivation. |
| 20. The authors may want to consider using a qualitative check-list, such as COREQ to align the manuscript with common practices in qualitative write-ups. | We thank the reviewer for this helpful suggestion. The manuscript has been aligned with the Consolidated Criteria for Reporting Qualitative Research (COREQ). A statement indicating adherence to COREQ has been added to the Methods section, and a completed COREQ checklist has been provided as Supporting Information (S3). We also clarified issues related to researcher reflexivity, data saturation, and trustworthiness to further enhance transparency and rigor.  *“This manuscript was prepared in accordance with the Consolidated Criteria for Reporting Qualitative Research (COREQ) checklist to enhance transparency and rigor in qualitative reporting.”*  *“Researcher characteristics and reflexivity*  *Interviews were conducted by a trained qualitative researcher with prior experience working with transgender communities and HIV research. The interviewer was familiar with the local context and community-based service delivery, which facilitated rapport and open discussion. Reflexive notes were taken throughout data collection and analysis to acknowledge and minimize potential researcher bias.”* |
| 21. Discussion does a nice job connecting the study’s findings with the literature. | Thank you for your very positive feedback. |
| 22. Minor but make sure to spell out abbreviations and acronyms (TG, COM-B, etc.) the first time they are used in the manuscript. | All abbreviations (e.g., TG, COM-B, IDIs, PrEP) have now been spelled out at first use throughout the manuscript. |
| 23. The manuscript needs additional proofreading, as there were several places with typos and missing words that hindered readability. | The manuscript has undergone careful proofreading, and typographical errors and missing words have been corrected. |
